# Complete Nucleotide Sequence of IncP-1β Plasmid pDTC28 Reveals a Non-Functional Variant of the *bla*_GES_-Type Gene

**DOI:** 10.1371/journal.pone.0154975

**Published:** 2016-05-06

**Authors:** Bingjun Dang, Daqing Mao, Yi Luo

**Affiliations:** 1 School of Environmental Science and Engineering, Tianjin University, Tianjin, China; 2 Ministry of Education Key Laboratory of Pollution Processes and Environmental Criteria, College of Environmental Science and Engineering, Nankai University, Tianjin, China; University of Manchester, UNITED KINGDOM

## Abstract

Plasmid pDTC28 was isolated from the sediments of Haihe River using *E*. *coli* CV601 (*gfp*-tagged) as recipient and indigenous bacteria from the sediment as donors. This plasmid confers reduced susceptibility to tetracycline and sulfamethoxazole. The complete sequence of plasmid pDTC28 was 61,503 bp in length with an average G+C content of 64.09%. Plasmid pDTC28 belongs to the IncP-1β group by phylogenetic analysis. The backbones of plasmid pDTC28 and other IncP-1β plasmids are very classical and conserved, whereas the accessory regions of these plasmids are diverse. A *bla*_GES-5_-like gene was found on the accessory region, and this *bla*_GES-5_-like gene contained 18 silent mutations and 7 missense mutations compared with the *bla*_GES-5_ gene. The mutations resulted in 7 amino acid substitutions in GES-5 carbapenemase, causing the loss of function of the *bla*_GES-5_-like gene on plasmid pDTC28 against carbapenems and even β-lactams. The enzyme produced by the *bla*_GES-5_-like gene cassette may be a new variant of GES-type enzymes. Thus, the plasmid sequenced in this study will expand our understanding of GES-type β-lactamases and provide insights into the genetic platforms used for the dissemination of GES-type genes.

## Introduction

The dissemination of antibiotic resistance genes (ARGs) has been regarded as a threat to public health. The vehicles used for the dissemination of ARGs are mainly conjugative plasmids, among which the IncP-1 group of plasmids may be the most efficient vehicles because of their broad-host-range properties and high transfer frequencies among Gram-negative bacteria. In view of this, it is important to analyze the ARGs contained by the IncP-1 plasmids. To date, many types of ARGs have been found to be located on IncP-1 plasmids, including the clinically important carbapenem resistance gene.

Carbapenems are considered the last resort option against certain multi-resistant pathogens [1,2]. The importance of carbapenems in treating multi-resistant pathogens has made carbapenem resistance a research hotspot [3–9]. The main mechanism encoding carbapenem resistance is the production of carbapenem-hydrolyzing class D β-lactamases (CHDLs) such as OXA-23, OXA-24 and OXA-58 [10–12]. Another mechanism that is less frequently encountered than CHDLs is Ambler class B β-lactamases–metallo β-lactamases (IMP, VIM, SIM and NDM) [10–12]. Some of the Ambler class A β-lactamases (IMI, GES and KPC) also have the ability to hydrolyze carbapenems. Most of the GES-type β-lactamases are extended-spectrum β-lactamases (ESBLs), whereas some GES variants such as GES-4, GES-5, and GES-6 gain their carbapenemase activity due to a Ser170 substitution of Gly170 [10–12]. Among the carbapenem hydrolyzing GES variants, GES-5 was found to be the most prevalent worldwide. Recently, the *bla*_GES-5_ gene has been increasingly detected in a variety of Gram-negative bacteria of clinical importance, including *Pseudomonas aeruginosa*, *Klebsiella pneumoniae*, *Acinetobacter baumannii*, *Escherichia coli* and *Serratia marcescens*, reflecting the rapid dissemination of this type of gene [10,13–15]. Therefore, the therapeutic challenges raised by the *bla*_GES-5_ gene in the treatment of bacterial infections are escalating.

The *bla*_GES-5_ gene is always found to be located on plasmids. *bla*_GES-5_-harboring plasmids have various sizes and belong to different incompatible groups, mostly IncQ or IncQ-related groups [15–17]. Plasmid pCHE-A, which was found in Canada, belongs to the IncQ group [18]. The plasmids pG5A4Y427, pG5A4Y201, pG5A4Y413 and pG5A4Y217, which were also found in Canada, belong to the MOB_Q1_ group [15]. The MOB_Q1_ group was derived from the IncQ group and has close relationships with the IncQ1 plasmid RSF1010. The *bla*_GES-5_ gene was also found to be contained by ColE1-like plasmids, such as plasmid pKP-M1144 [19]. Girlich et al. studied two *bla*_GES-5_-harboring plasmids (pRSB113 and pRSB115) that were isolated from the activated sludge of a wastewater treatment plant and found that these two plasmids did not belong to any of the IncP, IncQ, IncN, IncW, or IncA/C groups [20]. To date, there are no reports of the *bla*_GES_-_5_ gene or other *bla*_GES_ variants carried by the IncP-1 group plasmids. Here, we report the complete sequence of the IncP-1β plasmid pDTC28, which was isolated from river sediments and carries a new non-functional variant of the *bla*_GES_-type gene.

## Materials and Methods

### Sample Collection

Sediment samples were collected from the Sewage River of Tianjin with a grab sampler and then placed into sterile containers. The sample was immediately taken to the laboratory and stored at -20°C for subsequent experiments after sampling was completed. In this study no specific permissions were required for the sampling activities and we confirm that our study did not involve endangered or protected species.

### Source of Plasmid pDTC28

The tetracycline-resistant conjugative plasmid pDTC28 was isolated from the sediments of the Sewage River by filter mating assays using *Escherichia coli* CV601 (*gfp*-tagged, kanamycin and rifampicin resistant) as recipient and the sediment sample as donor [21]. The procedure for the filter mating experiments used in this study was as described in Heuer et al. with slight modifications [21]. An overnight culture of the *Escherichia coli* CV601 recipient strain was centrifuged at 4880 g for 5 min and washed twice with 0.85% sterile sodium chloride. Then, the pellet was resuspended in 2.5 ml Luria-Bertani (LB) broth. We resuspended 2 g of sediment sample in 18 ml LB broth in a 50 ml Erlenmeyer flask with five sterile glass balls (4 mm diameter). Then, the flask was placed on a rotary shaker (200 rpm) for 2 h. After shaking, 4 ml sediment solution was transferred into a 10 ml Eppendorf tube that contained 1 ml *Escherichia coli* CV601 recipient. The mixture of sediment solution and *Escherichia coli* CV601 recipient was centrifuged at 4880 g for 5 min. After centrifugation, the pellet was resuspended with 100 μl LB broth and then spotted onto a 0.22 μm sterile filter disk, which was then placed on an LB agar plate supplemented with 100 mg L^-1^ cycloheximide. The plate was placed upside down in an incubator at 37°C for 2 days. After incubation, the bacteria on the filter disk were resuspended with 2 ml 0.85% sterile sodium chloride solution. Then, the suspension was plated on selective agar plates containing tetracycline (10 mg L^-1^), kanamycin (50 mg L^-1^) and rifampicin (50 mg L^-1^). The cycloheximide (100 mg L^-1^) was also included in the selective agar plates to inhibit fungal growth. The transconjugants were further determined by green fluorescence resulting from the green fluorescence protein (GFP) gene. The *Escherichia coli* CV601 recipient culture was plated on the same selective plates as the controls. The sediment sample was also plated on the selective plates to rule out the possibility of green fluorescence bacteria from the environment. The isolated tetracycline-resistant transconjugants were subjected to susceptibility testing by the Kirby-Bauer disk diffusion method on Muller-Hinton Agar plates. According to the criteria of the Clinical and Laboratory Standards Institute (CLSI), the antimicrobial agents on the disks used in this study are as follows: ampicillin (10 μg), gentamicin (10 μg), streptomycin (10 μg), tetracycline (30 μg), ciprofloxacin (5 μg), sulfamethoxazole (300 μg), erythromycin (15 μg) and imipenem (10 μg). *Escherichia coli* ATCC25922 was used as a quality control strain. One transconjugant that conferred resistance to sulfamethoxazole and tetracycline, namely, DTC28, was selected for further analysis. The conjugative plasmid harbored by DTC28 was named pDTC28 and stored for complete genome sequencing.

### Conjugative Frequency of Plasmid pDTC28

To assess the conjugative frequency of plasmid pDTC28, liquid mating experiments were performed using *Escherichia coli* CV601 (pDTC28) as the donor strain and *Escherichia coli* J53 (azide and nalidixic acid resistance) as the recipient strain. For the liquid mating assay, overnight cultures of donor and recipient strains were centrifuged, washed and adjusted to an optical density of 0.6 at the wavelength of 600 nm (OD_600_) with LB broth. Then, 0.5 ml cultures of donor and recipient strains were mixed and brought up to a volume of 5 ml with LB broth. After incubation for 16 h at 37°C, transconjugants were selected on LB plates containing azide, nalidixic acid and tetracycline. The concentrations of antibiotics used for selecting transconjugants were described above. The conjugative frequency was determined by the following formula: Conjugative frequency = transconjugants (CFU/ml)/recipients (CFU/ml).

### Plasmid Sequencing and Bioinformatics

Plasmid DNA was extracted from the *E*. *coli* J53 transconjugants of plasmid pDTC28 using the Qiagen Plasmid Midi Kit (Qiagen Inc., Germany). A shotgun library was generated and then sequenced on an Illumina HiSeq 4000 sequencing system. Sequencing was performed by the Beijing Genomics Institute (BGI, Beijing, China). Sequencing reads were *de novo* assembled into contigs using the SOAPdenovo 2.04 software [22,23], followed by gap closure through a PCR and Sanger sequencing approach. The putative open reading frames (ORFs) were predicted using Glimmer 3.02 [24–26]. All ORFs were translated and aligned with different protein databases including NR (version: 20121005), KEGG (version: 59), COG (version: 20090331), SwissProt (version: 201206) and GO (version: 1.419).

### Phylogenetic Analysis and Visualization

Multiple alignments were carried out using the CLUSTALX program. Phylogenetic trees were constructed using iTOL (http://itol.embl.de/) [27] based on the neighbor-joining method. The accession numbers of plasmids used in the construction of phylogenetic trees are JX469829 (pB1), JX469826 (pB12), JX469828 (pRSB223), KP792998 (pBAM1), NC_019264 (pNB8c), AJ431260 (pB4), NC_016968 (pTB30), NC_019263 (pLME1), NC_008459 (pBP136), NC_004956 (pADP-1), AJ639924 (pB3), JX486125 (pRWC72a), JN106164 (pAKD1), KU238092 (pDTC28), JN106169 (pAKD18), JN106173 (pAKD31), JN106174 (pAKD33), JN106172 (pAKD29), NC_019312 (pKV29), AB852526 (pAAA83), NC_021077 (pBHB), JN106165 (pAKD14), JN106166 (pAKD15), AJ863570 (pB8), AJ564903 (pB10), AM048832 (pTP6), KC170279 (pMBUI8), NC_001735 (R751), JN106168 (pAKD17), KC170283 (pDS1), JQ432564 (pKS208), AM157767 (pQKH54), JQ432563 (pMBUI1), NC_025029 (pAKD4), NC_005793 (pEST4011), NC_019378 (pIJB1), CP002151 (pB5), CP002153 (pSP21), AJ744860 (pTB11), U05774 (RK2), NC_008357 (pBS228), JX469833 (pWEC911), JN106167 (pAKD16), JN106170 (pAKD25), JQ004407 (pHH3408), JQ004409 (pKS77), JQ004406 (pHH128), AM261282 (pKJK5), KC964607 (pMLUA4), KC964606 (pMLUA3), KC964605 (pMLUA1), JQ004408 (pHH3414), AY950444 (pMCBF1) and NC_025028 (pMCBF6). The circular map of plasmid pDTC28 was generated by Circos [28].

### Nucleotide Sequence Accession Number

The complete nucleotide sequence of pDTC28 was deposited in GenBank under accession no. KU238092.

## Results and Discussion

### General Structure of *bla*_GES-5_-harboring IncP-1β Plasmid pDTC28

**Fig 1:**
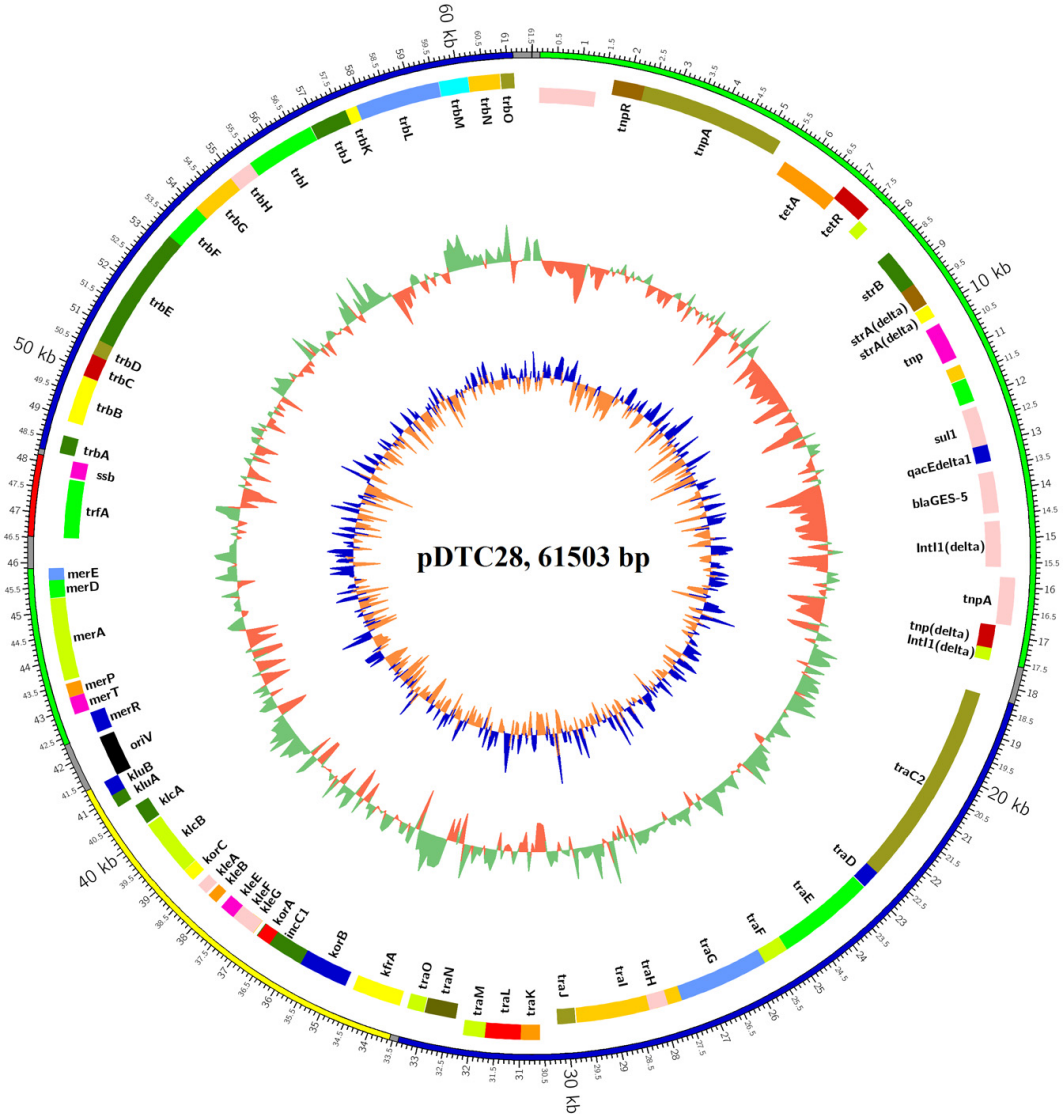
Circular map of plasmid pDTC28 (GenBank KU238092). The rings show from outside to inside the (i) position of different regions of plasmid pDTC28 (regions are color-coded to represent different functions: transfer region (blue); accessory region (green); replication region (red); stable inheritance and central control region (yellow)), (ii) position of predicted coding sequences in the clockwise direction, (iii) position of predicted coding sequences in the counterclockwise direction, (iv) GC content in a 100-bp window and (v) GC skew in a 100-bp window. The *oriV* region is marked with a black box.

**Fig 2:**
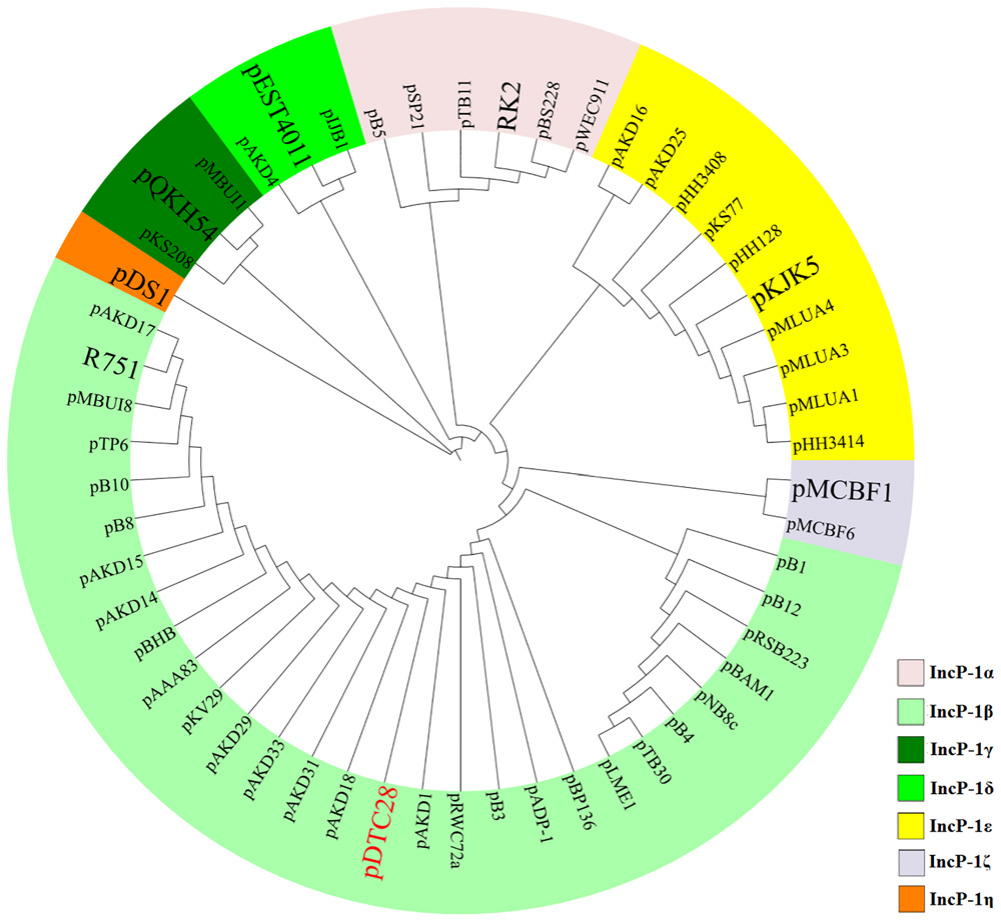
Phylogenetic relationships of plasmid pDTC28 with other IncP-1 plasmids. The amino acid sequences of the *trfA* genes from 54 IncP-1 plasmids were used to demonstrate the phylogeny of IncP-1 plasmids. The incompatibility subgroup of each clade is color-coded. The prototype of each subgroup is highlighted in large font.

The complete sequence of plasmid pDTC28 was 61,503 bp in length with an average G+C content of 64.09% (Fig 1). The overall sequence of plasmid pDTC28 can be divided into two main parts: the backbone region (positions 18291 bp-41377 bp, 46514 bp-61134 bp) and the accessory region (positions 157 bp-17566 bp, 42362 bp-45882 bp). Plasmid pDTC28 was found to belong to the IncP-1 group by sequence queries against the GenBank database. The IncP-1 plasmids were originally divided into two subgroups based on the sequence similarities with plasmid RP4 and R751 [29]. RP4 is the prototype of the IncP-1α group, whereas R751 is the prototype of the IncP-1β group. Currently, there are seven subgroups of IncP-1 plasmids including α, β, γ, δ, ε, ζ and η [30]. A phylogenetic tree was constructed using the *trf*A proteins of 54 IncP-1 plasmids including plasmid pDTC28 (Fig 2). The tree shows that plasmid pDTC28 belongs to the IncP-1β group and clusters with the IncP-1β prototype R751. The tree also shows that plasmid pDTC28 is slightly different from a smaller IncP-1β clade formed by plasmids pB1, pB12, pNB8c, pLME1, pB4, pTB30, pBAM1 and pRSB223.

**Fig 3:**
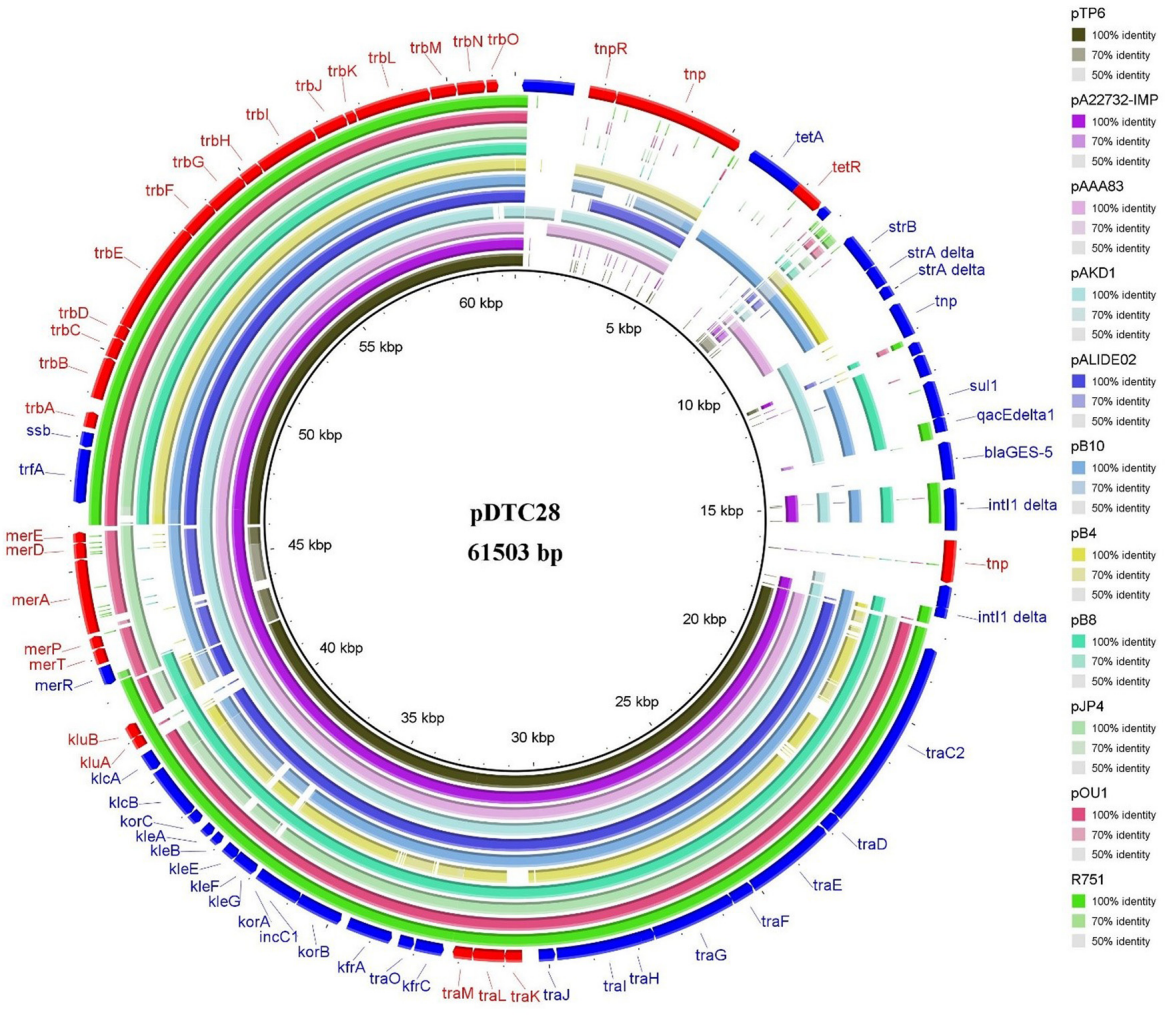
Pairwise comparisons between plasmid pDTC28 with other 11 IncP-1β plasmids. There are 12 rings in this map. From the innermost to the outermost, the rings represent the sequences of different IncP-1β plasmids: ring 1, plasmid pTP6 (AM048832); ring 2, plasmid pA22732-IMP (KJ588780); ring 3, plasmid pAAA83 (AB852526); ring 4, plasmid pAKD1 (JN106164); ring 5, plasmid pALIDE02 (NC_014911); ring 6, plasmid pB10 (AJ564903); ring 7, plasmid pB4 (AJ431260); ring 8, plasmid pB8 (AJ863570); ring 9, plasmid pJP4 (AY365053); ring 10, plasmid pOU1 (NC_005088); ring 11, plasmid R751 (NC_001735); ring 12, annotations of plasmid pDTC28.

There are several completely sequenced IncP-1β plasmids that are highly similar to plasmid pDTC28. Eleven of these sequenced plasmids, including the IncP-1β plasmid prototype R751, were selected from the GenBank database and then compared with plasmid pDTC28. Fig 3 shows the organizations of the 11 plasmids in comparison with the plasmid pDTC28. This figure demonstrates that the backbones of plasmid pDTC28 and these 11 sequenced plasmids are very classical and conserved, while the accessory regions of these plasmids are quite different from one plasmid to another. The backbone of plasmid pDTC28 (positions 18291 bp-41377 bp, 46514 bp-61134 bp) includes the replication region (positions 46514 bp-48122 bp), stable inheritance and central control region (positions 33559 bp-41377 bp), and transfer region (positions 18291 bp–33388 bp and 48236 bp-61134 bp), altogether constituting up to 61.3% of the total sequence.

The replication region of pDTC28, including the replication initiation protein gene *trfA* and the single-strand DNA binding protein gene *ssb*, shared 100% nucleotide similarity with the equivalent regions on plasmids pA22732-IMP (GenBank accession no.: KJ588780) [31], pAAA83 (GenBank accession no.: AB852526) [32], pALIDE02 (GenBank accession no.: NC_014911) [33], pUO1 (GenBank accession no.: NC_005088) [34], pB8 (GenBank accession no.: AJ863570) [35] and pTP6 (GenBank accession no.: AM048832) [36].

Plasmid pDTC28 contained two putative transfer regions (positions 18291–33388 bp and 48236–61134 bp) that comprised, respectively, 13 *tra* genes (*traC2*, *traD*, *traE*, *traF*, *traG*, *traH*, *traI*, *traJ*, *traK*, *traL*, *traM*, *traN* and *traO*) and 15 *trb* genes (*trbA*, *trbB*, *trbC*, *trbD*, *trbE*, *trbF*, *trbG*, *trbH*, *trbI*, *trbJ*, *trbK*, *trbL*, *trbM*, *trbN* and *trbO*). Mating experiments demonstrated that plasmid pDTC28 was self-transmissible at a relatively high frequency of (1.18±0.267) ×10^−2^ transconjugants per recipient between *E*. *coli* CV601 and *E*. *coli* J53, supporting that these two putative transfer regions are functional.

The stable inheritance and central control region of plasmid pDTC28 consists of the *incC1* gene, which is involved in plasmid active partitioning; the *kle* genes (*kleA*, *kleB*, *kleC*, *kleD*, *kleE*, *kleF* and *kleG*), which are involved in plasmid stable inheritance and can enhance the frequency of plasmid active partitioning; the *kor* genes (*korA*, *korB* and *korC*) and the *kfrA* gene, which are involved in transcriptional regulation; the *klc* genes (*klcA* and *klcB*), which are also involved in plasmid stable inheritance; and the *klu* genes (*kluA* and *kluB*), which may be involved in plasmid stability (post-segregational killing).

### Accessory Region of Plasmid pDTC28

The accessory region of plasmid pDTC28 contained a putative mercury resistance locus (positions 42362 bp-45882 bp), a putative class 1 integron (positions 12198 bp-15593 bp) harboring the *bla*_GES-5_-like gene, tetracycline resistance gene cassettes (positions 5580 bp-7535 bp) and aminoglycoside resistance gene cassettes (positions 8473 bp-9800 bp). This region was 21,031 bp in length and inserted into two loci, one between the *tra* region and *trb* region and the other between the *oriV* region and the *trfA* gene, altogether accounting for 34.2% of the total sequence.

### Mercury Resistance Locus

Plasmid pDTC28 possessed a putative mercury resistance locus (positions 42362 bp-45882 bp) for transporting mercury-derived compounds out of the bacterial cell. This mercury resistance locus contained the *merE* gene (inner membrane spanning proteins for transporting Hg^2+^ to the cytoplasm, where it is reduced by *merA*), the *merD* gene, the *merA* gene (mercuric reductase, enzymatic reduction of Hg^2+^ to Hg^0^), the *merP* gene (periplasmic Hg^2+^ scavenging protein), the *merT* gene (inner membrane spanning proteins for transporting Hg^2+^ to the cytoplasm, where it is reduced by *merA*) and the *merR* gene (regulatory protein acting as a transcriptional repressor or activator for regulating the overall expression of the *mer* operon) [37,38].

The *mer* locus is relatively conserved and prevalent among the IncP plasmids. This region of plasmid pDTC28 is almost identical to that of plasmid pA22732-IMP (99% nucleotide identity) and plasmid pAAA83 (100% nucleotide identity). As shown in Fig 3, this region of plasmid pDTC28 also shows high similarity to other IncP-1β plasmids, including plasmid pAKD1 (GenBank accession no.: JN106164) (95% nucleotide identity) [39], pJP4 (GenBank accession no.: AY365053) (94% nucleotide identity) [40] and pB10 (GenBank accession no.: AJ564903) (94% nucleotide identity) [41]. Plasmid pDTC28 was isolated from the sediments of the Sewage River in Tianjin. The Sewage River receives a large amount of wastewater of different origins, including domestic wastewater and industrial wastewater. Previous studies conducted by Shi et al. have demonstrated that the concentrations of mercury in the Sewage River sediments were extraordinarily high [42]. The emergence of the mercury resistance-encoding plasmid pDTC28 exemplifies the means by which environmental bacteria survive the high mercury selective pressure in the Sewage River sediments.

### Class 1 Integron Harboring the *bla*_GES-5_-like Gene in the Accessory Region

**Fig 4:**
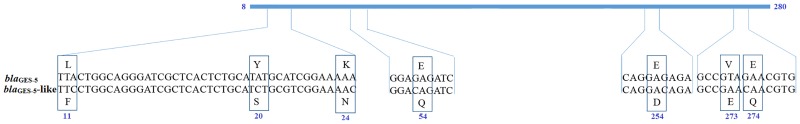
Sequence alignment of the *bla*_GES-5_-like gene of plasmid pDTC28 with the *bla*_GES-5_ gene from the *Escherichia coli* (GenBank accession no.: AY494717). The differences in amino acids between the products of the *bla*_GES-5_ and *bla*_GES-5_-like genes are highlighted in the blue box.

The accessory region of plasmid pDTC28 contains a putative class 1 integron. This integron contains a single gene cassette that shares 97% identity with the *bla*_GES-5_ gene with 100% query coverage. The *bla*_GES-5_ gene is believed to encode resistance to carbapenems. The GES-type enzymes possess the ability to hydrolyze broad-spectrum cephalosporins. Some variants of GES-type enzymes such as GES-2, -4, -5, -6, -11, and -14 have an enlarged spectrum of activity against carbapenems through amino acid substitutions [10]. Although the *bla*_GES-5_-like gene differs only in a few nucleotides with the canonical *bla*_GES-5_ gene, the gene did not confer resistance to carbapenems or even ampicillin in antibiotic susceptibility testing. Compared with the *bla*_GES-5_ gene from *E*. *coli* (GenBank accession number AY494717), the *bla*_GES-5_-like gene on pDTC28 contains 18 silent mutations and 7 missense mutations. As shown in Fig 4, the mutations resulted in 7 amino acid substitutions in GES-5 carbapenemase. Previous studies have shown that the Ambler positions 104 and 170 of GES-type β-lactamases are hot spots for amino acid substitutions [43]. Amino acids in these two sites play an important role in the interaction with β-lactams [43]. However, the substitutions in this study are not located in these two sites but rather mostly cluster at the two ends of GES-5 carbapenemase. Nonetheless, these substitutions have caused a loss of function of the *bla*_GES-5_-like gene cassette on plasmid pDTC28 against carbapenems and even β-lactams. The enzyme produced by the *bla*_GES-5_-like gene cassette may be a new variant of GES-type enzymes, and further study is needed to determine whether it has any biological role.

### Other Antibiotic Resistance Genes in the Accessory Region

Except for the *bla*_GES-5_-like gene, the accessory region of pDTC28 also contains other antibiotic resistance genes. The Δ*strA*-*strB* gene cluster (positions 8473 bp-9800 bp) was found to be located in the accessory region of plasmid pDTC28. The products of the *strA*-*strB* gene cluster could catalyze the transfer of a phosphate group to the aminoglycoside molecule [44]. However, like the *bla*_GES-5_-like gene, the *strA* gene on plasmid pDTC28 also has some differences in nucleotides with the normal *strA* gene. The *strA* gene on plasmid pDTC28 showed three mismatches and one insertion in comparison to the *strA* gene from plasmid RSF1010 (GenBank accession no.: M28829). The mismatches and insertion of the *strA* gene on plasmid pDTC28 have resulted in the disruption of the *strA* gene and the loss of function of the *strA*-*strB* gene cluster against streptomycin. According to the results of antibiotic susceptibility testing, plasmid pDTC28 did not confer resistance to streptomycin. This result is in correspondence with the sequence analysis. In addition to the Δ*strA*-*strB* gene cluster, plasmid pDTC28 also harbors the *tetA*-*tetR* gene cluster. The existence of the *tetA*-*tetR* gene cluster is consistent with the susceptibility results of plasmid pDTC28 to tetracycline.

In conclusion, we report the first complete sequenced IncP-1β plasmid that harbors the *bla*_GES-5_-like gene and the truncated *strA*-*strB* gene cluster. The *bla*_GES-5_-like gene is likely to encode a new variant of GES-type β-lactamase although it does not confer any known resistance phenotype. Therefore, we believe the plasmid sequenced in this study will expand our understanding of GES-type β-lactamases and provide insights into the genetic platforms used for the dissemination of GES-type genes.
